# Overexpression of Arginine Transporter CAT-1 Is Associated with Accumulation of L-Arginine and Cell Growth in Human Colorectal Cancer Tissue

**DOI:** 10.1371/journal.pone.0073866

**Published:** 2013-09-06

**Authors:** Ying Lu, Weimin Wang, Junchen Wang, Chunzhang Yang, Huiming Mao, Xuelian Fu, Yanling Wu, Jingping Cai, Junyi Han, Zengguang Xu, Zhengping Zhuang, Zhongmin Liu, Hai Hu, Bingguan Chen

**Affiliations:** 1 Clinical Translational Medical Research Center, Shanghai East Hospital, Tongji University School of Medicine, Shanghai, PR China; 2 Department of Surgery, Changzheng Hospital, Second Military Medical University, Shanghai, PR China; 3 Surgical Neurology Branch, National Institute of Neurological Disorders and Stroke, National Institutes of Health, Bethesda, Maryland, United States of America; National Cancer Institute (INCA), Brazil

## Abstract

We previously showed that L-arginine (Arg) accumulates in colorectal cancer tissues. The aim of this study was to investigate the mechanism by which Arg accumulates and determine its biological significance. The concentration of Arg and Citrulline (Cit) in sera and tumor tissues from colorectal cancer (CRC) patients was analyzed by high-performance liquid chromatography (HPLC). The expression of Arg transporters was analyzed by quantitative reverse transcription polymerase chain reaction (qRT-PCR) and immunohistochemical analysis of tissue microarray. We also transfected the colon cancer cell line HCT-116 with siRNA specific for the Arg transporter CAT-1 and measured the induction of apoptosis by flow cytometry and cell proliferation by MTT assay. Consistent with our previous results, serum Arg and Cit concentrations in colorectal cancer patients were significantly lower than those in normal volunteers, while Arg and Cit concentrations in colorectal cancer tissues were significantly higher than in matched adjacent normal colon tissues. Quantitative RT-PCR showed that the *CAT-1* gene was highly overexpressed in 70.5% of colorectal cancer tissue samples relative to adjacent normal colon tissues in all 122 patients with colorectal cancer. Immunohistochemical analysis of tissue microarray confirmed that the expression of CAT-1 was higher in all 25 colorectal cancer tissues tested. CAT-1 siRNA significantly induced apoptosis of HCT-116 cells and subsequently inhibited cell growth by 20–50%. Our findings indicate that accumulation of L-Arg and Cit and cell growth in colorectal cancer tissues is associated with over-expression of the Arg transporter gene CAT-1. Our results may be useful for the development of molecular diagnostic tools and targeted therapy for colorectal cancer.

## Introduction

Colorectal cancer (CRC) is one of the leading causes of cancer-related deaths in the United States and China despite improvements in treatment over the last several years [1], [2]. The treatment options for CRC include surgery, chemotherapy, radiotherapy, and targeted therapies, among which surgery remains the most effective. However, even with comprehensive treatment the prognosis is still poor for patients with Dukes stage D disease, with an overall 5-year survival rate of 6.6%–11.9%. With an improved understanding of the molecular pathology of cancer, newly developed targeted therapy combined with 5-FU and oxaliplatin-based chemotherapy has demonstrated improved outcome in metastatic CRC (mCRC) patients. However, only approximately 20% of mCRC cases respond to current targeted therapy options [3].

Currently approved targeted therapeutic reagents for use in mCRC include Bevacizumab (Avastin™, Genentech/Roche, CA, USA), a monoclonal antibody targeted to vascular endothelial growth factor (VEGF) and cetuximab (Erbitux™, Imclone Systems, NJ, USA) or panitumumab (Vectibix™, Amgen, CA, USA), monoclonal antibodies targeted to epidermal growth factor receptor (EGFR). The tyrosine kinase inhibitors (TKIs), such as erlotinib and gefitinib, are another class of reagents targeted to EGFR. Bevacizumab is commonly used in combination with standard chemotherapeutic agents (e.g., 5-FU) as a first-line treatment for patients with mCRC and improves the overall survival of these patients by approximately 5 months. However, side effects such as hypertension, anorexia, proteinuria, and gastrointestinal perforation have limited its application in some cases. EGFR is directly involved in cell proliferation and metastatic progression through both RAS/RAF/MAPK and phosphatidylinositol 3-kinase (PI3K) signaling pathways. The effect of anti-EGFR therapy depends on whether the tumor has a KRAS mutation; anti-EGFR therapy is not effective for patients with a mutation in codon 12 or 13 of KRAS. Therefore, a great effort is underway to study biomarkers for CRC and develop novel treatments in order to increase the 5-year survival rate and improve the overall quality of life for patients with this disease [1].

Several studies have shown elevated levels of polyamines and altered levels of rate-limiting enzymes involved in both biosynthesis and catabolism in colon cancer and several other cancers. There is evidence that tumor growth absolutely requires polyamines for cancer cell proliferation [4], therefore the polyamine pathway is recognized as a rational target for chemoprevention and chemotherapeutics [4], [5], [6]. Polyamines are produced by the action of ornithine decarboxylase (ODC) on ornithine that is produced by catabolism of L-arginine (Arg) by arginases that are overexpressed in cancer cells [4], [7], [8]. Consistent with this biosynthesis pathway, several lines of evidence have demonstrated that Arg is necessary for cancer development and progression [9], [10], [11], [12], [13], [14]. Both *in vitro* and *in vivo* studies have demonstrated that Arg is required for cancer cell proliferation, especially when endogenous Arg synthesis is blocked because of deficient argininosuccinate synthetase (ASS) expression [10], [11], [12], [13]. For tumor maintenance cancer cells overexpress the enzyme endothelial nitric oxide synthetase (eNOS), which consumes large amounts of Arg [15], [16]. Because of greatly accelerated Arg metabolism in cancer cells, Arg deprivation treatment has been developed to treat cancers that are ASS negative, such as hepatic carcinoma, renal cell carcinoma, and prostate cancer [10], [11], [12], [13].

When Arg is catabolized by NOS, the co-product of the NO pathway, Cit, can be recycled by ASS and argininosuccinate lyase (ASL) to synthesize Arg endogenously through the citrulline-NO or arginine-citrulline pathway [17], [18], [19]. Although the synthesis of Arg from Cit occurs at a low level in many cells, intracellular Arg synthesis can markedly increase under certain physiological or pathological circumstances that affect the homeostasis of circulating or intracellular Arg and Cit. Therefore, the effect of Arg deprivation treatment on cancer depends on whether the endogenous synthesis pathway is deficient. Early studies showed that human lung and colon carcinomas were almost always positive for ASS [20]. Moreover, disturbance of L-Arg bioavailability is associated with many diseases, including heart failure, immune deficiency, and cancer progression [9], [21], [22], [23].

In the study of cancer immunology, tumor-infiltrating lymphocytes, macrophages, and dendritic cells have been found to be functionally deficient in cancer tissues due to low Arg availability in the tumor microenvironment [18], [21], [24]. Based on these experimental results a few groups initiated Arg supplementation treatment for cancer patients [25]. However, there is controversy over the role of Arg supplementation or Arg deprivation in cancer treatment, largely because there are no direct data on the precise bioavailability of Arg in the tumor microenvironment, especially in CRC. To clarify this issue, we developed a method to determine the Arg level in blood and CRC tissue [26], [27]. In our preliminary studies we observed low concentrations of Arg and its metabolite Cit in the sera of CRC patients and higher concentrations of Arg and Cit in the cancer tissues. Here, we further determined the availability of Arg in the tumor microenvironment and investigated the mechanism underlying the increased intracellular Arg level by analyzing the expression of Arg transporters and endogenous Arg synthesis enzymes ASS and ASL in CRC tissues. Our results indicated that Arg metabolism is accelerated in CRC and identify the Arg transporter SLC7A1 as a potential molecular target for CRC therapy.

## Methods

### Samples and HPLC

Serum, tumor tissue, and adjacent normal colon tissue were simultaneously obtained from surgical samples of CRC patients. A total of 122 paired tumor tissues and adjacent normal colon tissue samples were collected from 122 CRC patients (79 males and 43 females) with a mean age of 60±11 years (ranging from 37 to 79 years) from the Department of Surgery at Shanghai Changzheng Hospital. The patients included 27 with sigmoid flexure cancer, 5 with colon descending cancer, 46 with rectal cancer, 16 with colon ascending cancer, and 28 with colon transversal colon cancer. All patients were pathologically diagnosed as adenocarcinoma (Table 1). The study was approved by the Institutional Review Board of Tongji University School of Medicine, and written informed consent was obtained from all individuals. Tissue samples for determining the expression of Arg transporters were stored at −80°C until analysis. Among 122 paired samples we collected 30 paired tissue samples and serum samples separately for HPLC to further determine the concentration of Arg and Cit. We were unable to test all individuals due to limited sample availability. Sera from 28 healthy volunteers who received annual health check and had no evidence of diseases were collected as a control. The demographic characteristics of 28 healthy volunteers and 30 colorectal cancer patients are shown in Table 2.

**Table 1:** Clinical data for colorectal cancer study participants.

Gender		Male	Female	Total	%
n		79	43	122	
Age(y)		61.8±10.9	57.7±11.9		
Location:					
	Ascending	10	6	16	13
	Transverse	18	10	28	23
	Descending	3	2	5	4
	Sigmoid	18	9	27	22
	Rectum	30	16	46	38
Stage:					
	I	6	4	10	8
	IIA	15	7	22	18
	IIB	12	11	23	19
	IIIA	13	3	16	13
	IIIB	16	11	27	22
	IIIC	10	4	14	11
	IV	7	3	10	8
Pathology:					
	Gx	19	7	26	21
	G1	0	0	0	0
	G2	34	17	51	42
	G3	26	19	45	37
	G4	0	0	0	0

**Table 2:** Demographic characteristics of individuals for serum analysis.

Group	Age(year)	Gender	
		Male	Female
CRC (n = 30)	59.26±12.33	16	14
Control (n = 28)	57.5±8.60	15	13

In terms of age and gender, there was no significant difference between two groups (*P*>0.5).

The levels of L-Arg and L-Cit in serum and tissue were determined by RP-HPLC using ultraviolet detection as we reported recently [24], [25]. The tumor and normal tissue were precisely weighed, and 0.1 g of tissue was homogenized in 0.5 ml of trichloroacetic acid (0.1 g/ml) in an ice bath. The homogenates were transferred into Eppendorf centrifuge tubes and analyzed similarly to the serum samples. The analytic content was calculated using the standard curve or regressive equation. The levels of L-Arg and L-Cit in sera were expressed as µmol/L and the levels of L-Arg and L-Cit in tissues were expressed as µg/g.

### Quantitative Reverse Transcription Polymerase Chain Reaction (qRT-PCR*)*

RNA was isolated from colorectal tissues using TRIzol (Invitrogen, Carlsbad, CA). The cDNA was immediately reverse transcribed from isolated RNA with SuperScript III First-Strand Synthesis System (Invitrogen, Carlsbad, CA). Quantitative RT-PCR was performed using the SYBR Green PCR kit (Qiagen, Valencia, CA). The primers used for each arginine transport gene are listed in table S1. GAPDH was used as an internal control for comparison of the data. Quantitative PCR was performed using the Applied Biosystems 7900HT (ABI, Foster City, CA), and the comparative Ct method was used to assess relative changes in mRNA levels between two samples.

### Tissue Microarray

Paraffin-embedded tissue microarray (CO951, US Biomax, Rockville, MD) was used for immunohistochemistry analysis as previously described [28]. Tissue array was processed with heat-induced antigen retrieval using 10 mM sodium citrate buffer, pH 6.0. The array was then stained with CAT-1 antibody (Abcam, Cambridge, MA) and visualized using a DAB staining kit.

### Cell Culture

The human colon cancer cell line HCT 116 was purchased from The Cell Bank of Chinese Academy of Sciences and cultured in a humidified, 5% CO_2_ atmosphere at 37°C. The culture medium used was McCOY’s 5A Medium (Sigma, St. Louis, MO) containing 10% v/v heat-inactivated fetal bovine serum (FBS).

### Transfection of Small Interfering RNA (siRNA)

Non-targeting siRNA (siNT) and siCAT-1 (Santa Cruz, Dallas, TX) were used at a concentration of 10 nM and transfected into HCT 116 cells using Lipo 2000 reagent (Invitrogen, Carlsbad, CA) following the manufacturer’s instructions. After 24 h, cells were seeded onto chambered slides or 24-well plates, and allowed to grow for another 24–48 h prior to RNA isolation or the start of experiments. Knockdown of gene expression was confirmed by q-PCR.

### Flow Cytometry and Cell Proliferation Analysis

The number of apoptotic cells was determined using Anti-annexin V mAb (BD, Franklin Lakes, NJ) and analyzed by flow cytometry as described in the literature [29]. Cell proliferation was determined by MTT assay kit (R&D Systems, Minneapolis, MN) as instructed by the manufacturer.

### Statistical Analysis

All statistical analyses were performed using SPSS 14.0 software. The concentrations of Arg and Cit are expressed as the mean ± standard deviation (mean ± SD). Differences in the average values between groups were assessed using the Student’s t-test. A *P*-value less than 0.05 was considered statistically significant.

## Results

### Serum Levels of Arg and Cit were Lower in Colorectal Cancer Patients than in Normal Individuals

To extend our previous findings we measured serum Arg and Cit in another 30 CRC patients and confirmed that serum Arg and Cit concentrations were significantly lower in patients with CRC than in normal individuals (Table 3). The serum Cit concentration was 39.22±13.33 µmol/L in CRC patients, compared with 86.27±23.54 µmol/L in normal controls, and the corresponding serum level of Arg was 84.83±26.18 µmol/L and 117.72±40.19 µmol/L, respectively. These results are consistent with those of our preliminary study.

**Table 3:** Serum concentration of citrulline and arginine in normal volunteers and patients with colorectal cancer (mean ± SD).

Amino acid	Normal control(µmol/L)	n	Cancer patients(µmol/L)	n
Citrulline	86.27±23.54	28	39.22±13.33**	30
Arginine	117.72±40.19	28	84.83±26.18*	30
Cit/Arg	0.81±0.29	28	0.48±0.21**	30

** Compared with normal subjects p<0.001;

* Compared with normal subjects P<0.005.

### Accumulation of Arg and Cit in Cancer Tissue of CRC Patients

**Figure 1:**
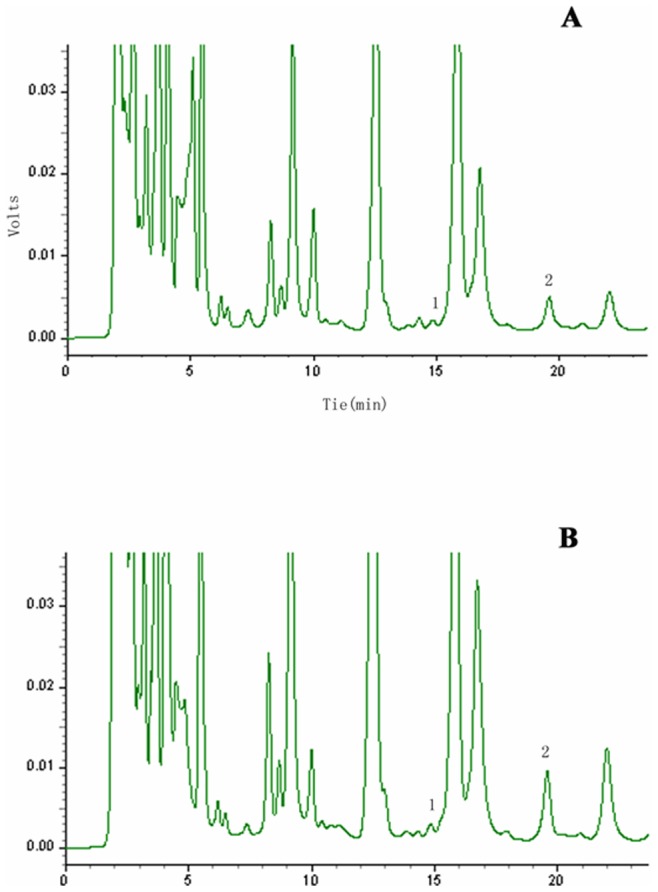
Chromatogram of HPLC for L-citrulline and L-arginine in colorectal tissues. The upper panel (a) shows the result from paired adjacent normal sigmoid flexure tissue in a patient with sigmoid colon cancer. The lower panel (b) shows the result from sigmoid flexure cancer tissue in the same patient. The individual marked peaks (1) and (2) represent L-citrulline and L-arginine respectively.

**Figure 2:**
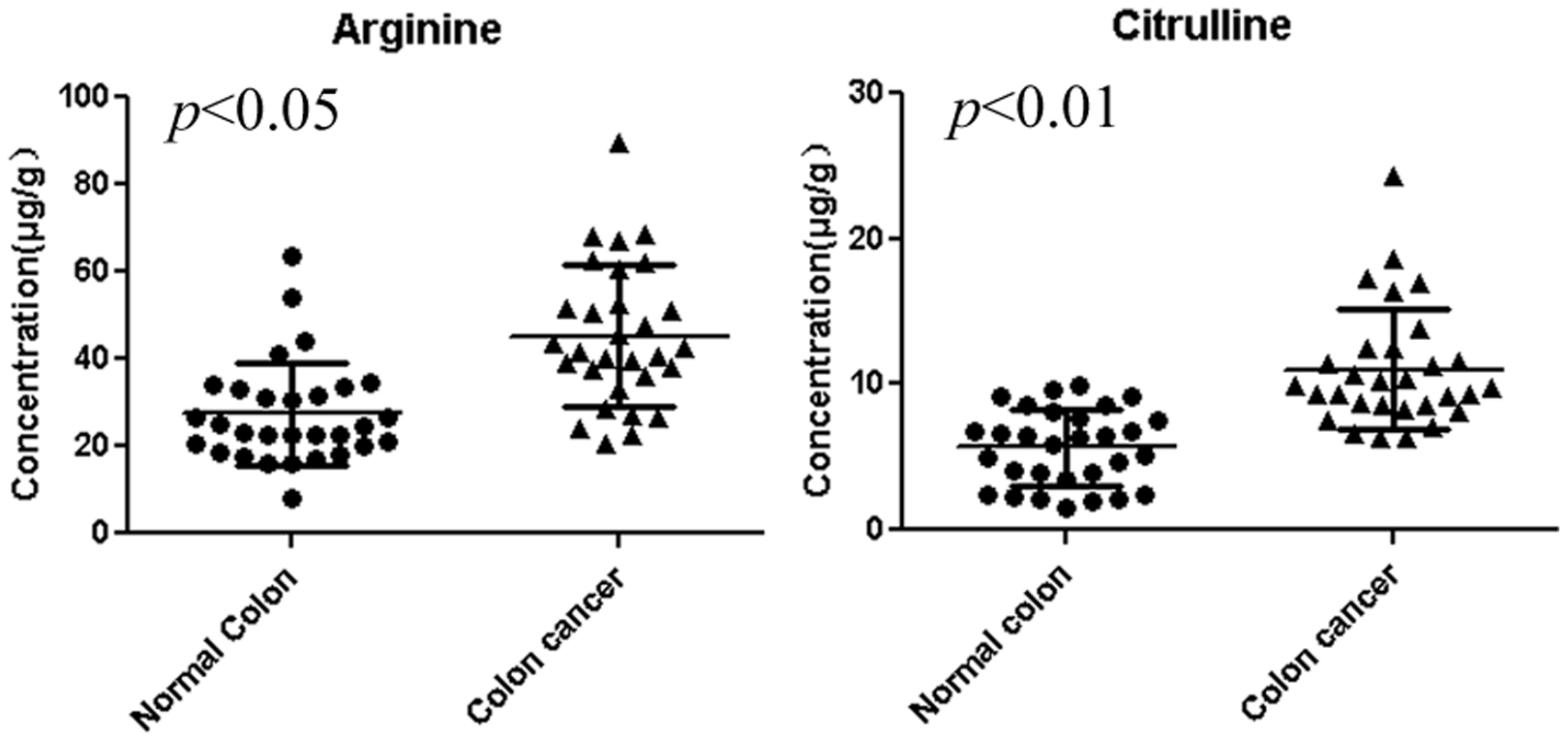
Concentration of Arg and Cit in colorectal cancer tissues and matched normal colon tissues from 30 colorectal cancer patients. Concentrations of both Arg and Cit were significantly higher in colorectal cancer tissues compared with paired adjacent normal colon tissues (*P*<0.05 and *P*<0.01 respectively). The detailed concentrations and statistical analyses are shown in Table 4.

To expand on our previous findings of higher availability of Arg and Cit in CRC tissues we investigated the reason for the reduced concentration of Arg and Cit in the sera of CRC patients. Based on the requirement for Arg in cancer cell proliferation we expected a higher consumption of Arg in CRC tissues. Therefore, we measured Arg and Cit levels in CRC tissues and found a dramatic accumulation of Arg and Cit (Figure 1). Both Arg and Cit concentrations were two-fold higher in the tumor tissue than in normal colon tissue from the same patient. These results were reproducible in 30 patients with CRC. The Arg level in colorectal cancer tissues was 45.26±17.59 µg/g compared with 27.34±11.59 µg/g in normal colon tissues (*P*<0.005), while the Cit level in CRC tissues was 11.01±4.16 µg/g compared with 5.60±2.61 µg/g in normal colon tissue (*P*<0.01, Table 4 and Figure 2). These results indicate that the bioavailability of Arg and Cit is elevated in colon cancer tissues, which may contribute to the lower levels of both Arg and Cit in the sera of CRC patients, as previously reported [26].

**Table 4:** The concentration of citrulline and arginine in colorectal cancer tissues and paired adjacent normal colon tissues (mean ± SD).

Amino acid	Normal tissues (µmol/L)	n	Cancer tissues(µmol/L)	n
Citrulline	5.60±2.61	30	11.01±4.16**	30
Arginine	27.34±11.59	30	45.26±17.59*	30
Cit/Arg	0.23±0.11	30	0.25±0.07	30

** Compared with normal tissue P<0.001;

* Compared with normal tissue P<0.005.

### Overexpression of CAT-1 in CRC Tissues by qRT-PCR Analysis

The accumulation of Arg and Cit in CRC tissues stimulated an investigation into the identity of the relevant transporter and the possibility that it might regulate cancer progression. The cationic amino acids transporters (CATs), a subfamily of the solute carrier family 7 (SLC7A), are the main transporters responsible for Arg influx. There are four confirmed transport proteins for cationic amino acids, CAT-1 (SLC7A1), CAT-2A (SLC7A2A), CAT-2B (SLC7A2B), and CAT-3 (SLC7A3). The function of human SLC7A3 and SLC7A4 is unknown, while the HATs 4F2hc/y+LAT1 and 4F2hc/y+LAT2 (SLC3A2/SLC7A7 and SLC7A6) accept L-type cationic and neutral amino acids. We measured the expression of genes encoding these arginine transporters in CRC and paired adjacent normal cancer tissues from 122 patients with CRC using qRT-PCR.

**Figure 3:**
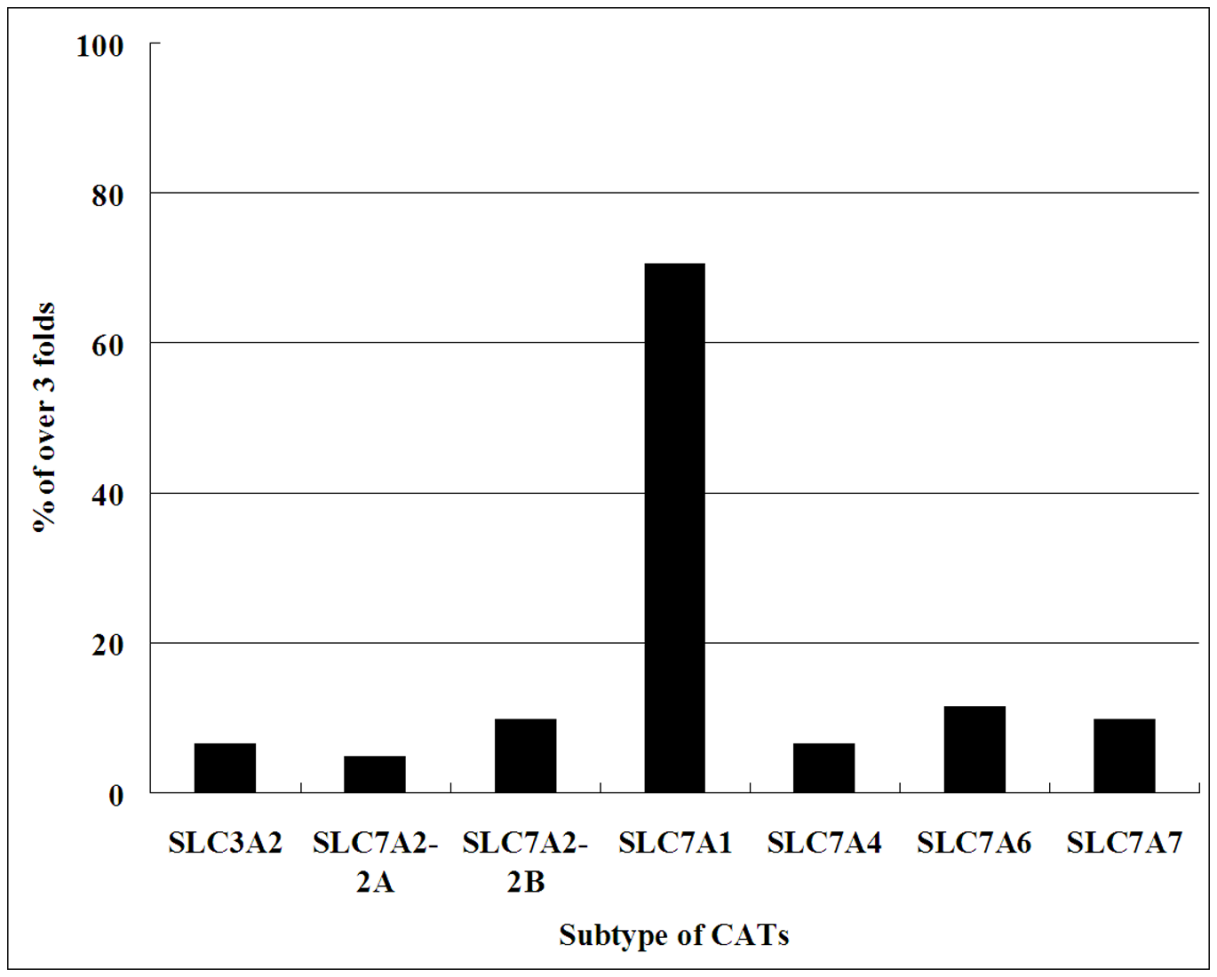
Overexpression of *CAT* mRNA in tumor relative to normal colon. The expression of CAT mRNA in colorectal cancer tissues was measured by qRT-PCR, and overexpression was defined as at least 3-fold higher expression than that in normal colon tissue. The figure shows the percentage of samples with overexpression (>3 fold) of individual arginine transporter genes among122 CRC tissue samples. The *CAT-1* gene was overexpressed in 86 of 122 (70.5%) CRC tissues.

As shown in Figure 3, when more than 3-fold over-expression was set as the cut-off value, *CAT-1* gene expression was elevated in CRC tissues in 86 of 122 patients (70.5%), in whom the expression level of CAT-1 in CRC tissues was 3.6- to 181-fold higher than in normal colon tissues, whereas expression of SLC7A2A and SLC7A2B was elevated in only 6/122 and 12/122 (4.9 and 9.8%) patients respectively. We also observed that SLC7A4 expression was elevated in 8/122 (6.6%) patients. Expression of SLC3A2, SLC7A6, and SLC7A7 was elevated in 8, 14, and 12 of the 122 patients respectively (6.6%, 11.5%, and 9.8%) (Figure 3). Our results indicate that overexpression of CAT-1 may be a major contributor to Arg accumulation in CRC tissues.

### Increased CAT-1 Protein Expression in CRC Tissues

**Figure 4:**
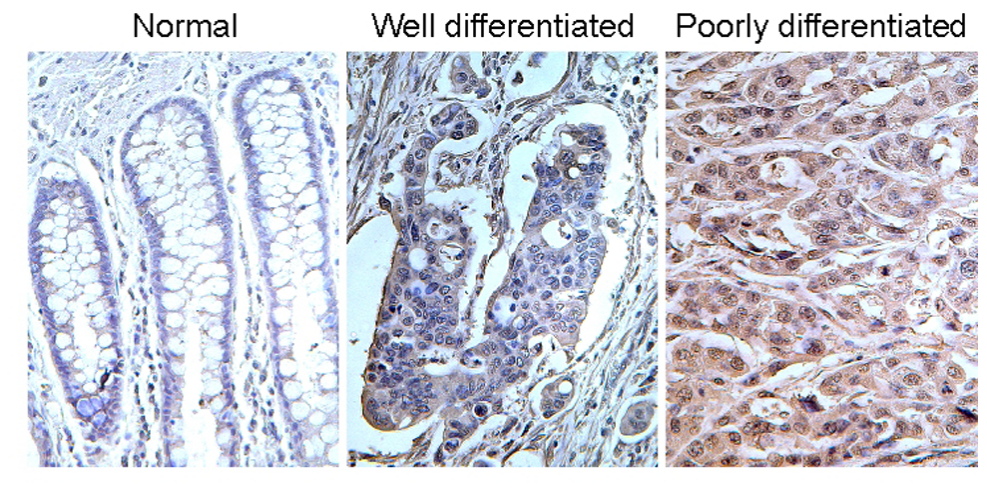
CAT-1 protein expression in colorectal cancer tissues by tissue microarray. The CRC tissue microarray (TMA) was stained with CAT-1 antibody and visualized using a DAB staining kit. The density of CAT-1 expression in the normal colon, well differentiated CRC, and poorly differentiated CRC samples from TMA was compared. The images were taken at 40× magnification.

To confirm the overexpression of CAT-1 in CRC tissues we further determined the CAT-1 protein level by immunohistological staining of 25 colon cancer samples in a tissue microarray (Figure 4). The expression of CAT-1 protein was weak in normal adjacent colon but elevated in colon adenocarcinomas. The CAT-1 expression level correlated with the differentiation grades of tumors; we found moderately increased levels of CAT-1 in well-differentiated colon adenocarcinoma (n = 8), and extensively up-regulated CAT-1 in poorly-differentiated specimens (n = 17). These results confirmed an increase in CAT-1 protein level in CRC tissues, consistent with the qRT-PCR findings.

### CAT-1 RNAi Inhibited the Growth of CRC Cells

**Figure 5:**
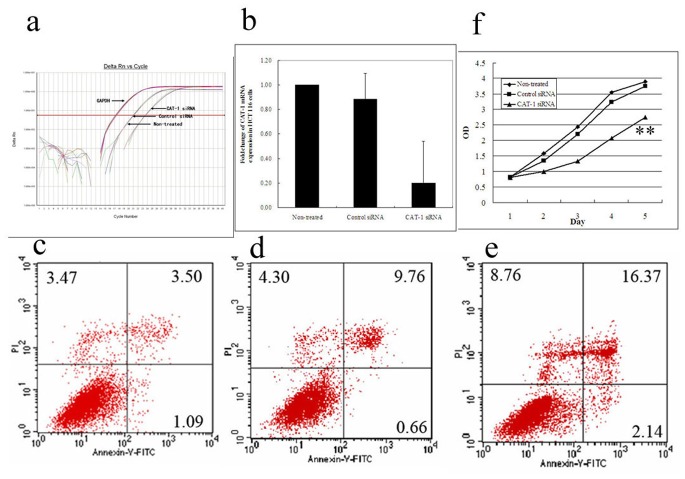
CAT-1 RNAi suppressed the cell growth of colorectal cancer cells. The colon cancer cell line HCT-116 was cultured *in vitro* in 6-well plates and were transfected with individual siRNAs, followed by analyzing the CAT-1 expression by qRT-PCR (A,B), apoptosis 72 hours after siRNA transfection by flow cytometry (C–E), and cell growth by MTT assay (F). CAT-1 siRNA successfully knocked down approximately 80% of CAT-1 expression (B). Compared with no treatment (C) and control siRNA (D), CAT-1 siRNA induced apoptosis by up to 16.37% (E). CAT-1 siRNA (triangle) significantly reduced cell viability of HCT116 colon cancer cells compared with no treatment and control siRNA transfection (F). The results were reproducible for three independent experiments. ** indicates *P*<0.01.

Based on the findings of Arg accumulation and higher CAT-1 expression in CRC tissues we further hypothesized that CAT-1 expression may correlate with cancer cell proliferation and subsequent cancer progression. We therefore performed an *in vitro* assay to study the effect of CAT-1 suppression by RNAi in colon cancer cells. As shown in Figures 5A and B, CAT-1 siRNA successfully knocked down (80% reduction determined by qRT-PCR) the expression of CAT-1 in HCT 116 colon cancer cells, consistent with the results in breast cancer cells [30]. Transfection with CAT-1 siRNA decreased tumor cell viability, promoted apoptosis (Figure 5C–E), and therefore inhibited the cell growth *in vitro* by 20–50% (Fig. 5F). Our results suggest that the Arg metabolism pathway might be a potential molecular target for CRC therapy.

## Discussion

In a continuation of our previous study [26], [27], we further examined the serum levels of Arg and Cit in CRC patients and their bioavailability in CRC tissue. We consistently demonstrated a decreased serum level of Arg and Cit in CRC patients and accumulation of both Arg and Cit in CRC tissues. Our results suggest that lower bioavailability of tumor infiltrating lymphocytes and tumor-related immune cells might not be related to Arg concentration in the cancer microenvironment, but rather might be related to the tumor cells’ metabolic characteristics and their ability to take up Arg. The concomitant high intracellular levels of Arg and Cit could be due to acceleration of intracellular synthesis pathways because Arg and Cit can be mutually metabolized by intracellular ASS/ASL and NOS. Recent studies showed that tumor endothelial cells express high levels of NOS, which promotes lymphatic metastasis and angiogenesis [15], [16], [31]. Thus, the elevated Cit concentration in the cancer tissues of patients in our study could be due to accelerated Arg metabolism by NOS, although the transporter in the cancer tissue and its specific activity for Cit remain unclear.

The intracellular synthesis Arg from Cit in the Arg-Cit pathway requires two enzymes, ASS and ASL. Several groups have reported a deficiency in endogenous Arg synthesis in melanoma, hepatic carcinoma, renal cell carcinoma, and prostate cancers as a result of deficient ASS [10], [11], [12], [13], [20]. Some other human cancers, including sarcomas, invasive breast carcinoma, and renal cell carcinoma, have been shown to be ASS-deficient in some studies, but human lung and colon carcinomas were almost always positive for ASS [20]. We consistently demonstrated increased expression of ASS and ASL in CRC tissue compared with normal colon tissue by immunohistochemistry, suggesting that the endogenous synthesis of Arg in CRC cells may be intact, and even enhanced, rather than deficient (Figure S1 and S2). The enhanced expression level of ASS and ASL in CRC may be partly responsible for the high Arg levels observed in cancer tissues.

It is known that the intracellular concentration of Arg is largely affected by the activity of Arg transporters in which the cationic amino acid transports (CATs) are the main transporters for Arg influx [32], [33], [34]. The accumulation of Arg in CRC cells may be caused by increased influx from extracellular interstitial pools through Arg transports. In an early *in vitro* study, increased L-Arg transport through the Na(+)-independent y+ system was observed in CRC cells, whereas in the presence of epidermal growth factor (EGF) and transforming growth factor alpha (TGFα) stimulation L-arginine uptake could occur through the Na(+)-dependent transporter [14]. Therefore, we screened the expression of all cationic amino acid transports in CRC tissues using qRT-PCR and revealed that CAT-1 was expressed at a higher level in CRC tissues than in normal colon tissues. Another study showed that changes in CAT-1 mRNA levels might not necessarily affect CAT-1 protein levels [35]. However, our experiments consistently showed overexpression of both CAT-1 mRNA and protein in CRC tissues. Although CAT-2 is important for Arg transport, especially for NO production during macrophage activity [35], we did not find any evidence for this in CRC tissues. This difference may reflect organ or cell specificity and different requirements for cellular activity. A recent *in vitro* study showed that CAT-1 plays a role in Arg uptake and survival of breast cancer cells, and even in NO production [30]. An early tissue transcriptome study suggested that human CAT-1 is almost ubiquitously expressed, but highly expressed only in colorectal cancer cells, early erythroid cells, endothelial cells, and CD34 stem cells [36].

Although it remains unclear why cancer cells primarily use CAT-1 for Arg metabolism, several lines of evidence may provide clues. First, CAT-1 can be upregulated by several factors in the tumor microenvironment, such as polyamines, pathologic stress, signals for rapid division, and proinflammatory cytokines that also play roles in cancer development and progression [32], [37], [38], [39]. Second, despite its almost ubiquitous presence, CAT-1 expression is highly regulated genetically. In adult normal hepatocytes CAT-1 is not expressed because of high expression levels of the suppressive microRNA, miR-122 [40]. However, colon epithelial cells express very low levels of suppressive miR-122 [41], resulting in higher CAT-1 expression. In CRC cells miR-122 was even down-regulated, indicating a loss of control of CAT-1 expression [42]. Third, although CAT-1 protein on the cellular membrane mediates both influx/efflux and exchange of its substrates, arginine, lysine, and ornithine, between intracellular and extracellular pools, differential expression of CAT-1 protein on the plasma membrane of different organelles within the cells may regulate these amino acid pools in different organelles [32]. Intracellular Arg is known to be one of the most important amino acids in activation of the mechanistic target of rapamycin (mTOR), especially the mTORC1 signaling pathway that promotes tumorigenesis, cell survival, and proliferation [42]. The activation of mTORC1 requires the translocation of mTORC1 from a poorly characterized cytoplasmic location to the lysosomal surface in the presence of amino acids [43], [44]. Therefore, the exact pool of amino acids in the organelle of cytoplasm or lysosome is important for amino acid sensing and subsequent mTORC1 signaling. In addition, CAT-1 protein on the plasma membranes plays an important role in intracellular compartmentalization and channeling of Arg to distinct metabolic pathways within the cytoplasm [32]. Taken together, these findings suggest that the subcellular location of CAT-1 may contribute to the pool of Arg in different organelles within the cells. Nevertheless, the results of Arg accumulation and overexpression of CAT-1 in CRC tissues presented here warrant further clarification of the intracellular distribution of CAT-1 in CRC cells and its biological significance in tumorigenesis.

Furthermore, our *in vitro* study demonstrated that knock-down of CAT-1 in CRC cells induced apoptosis and inhibited cell growth, suggesting that CAT-1 may be a unique molecular biomarker and therapeutic target of CRC. Early studies indicate that transport of certain amino acids is a general feature in neoplastic cells; in fact transport of 2-deoxy-D-glucose has been translated into the clinical application of PET-CT [45]. By a similar principle our findings may potentially translate into clinical applications, such as Arg-based radiodiagnosis or radiotherapy and CAT-1–based molecular target therapy. Further detailed study of the molecular mechanism of Arg transport in neoplasm cells is warranted since many unresolved issues remain, such as the regulation and distribution of CAT expression in cancer cells.

## Supporting Information

Figure S1 **Strong expression of ASS in colon carcinoma tissue as determined by immunohistochemical staining.** The samples are from matched tissue specimens: (a and b) cancer tissue, (c and d) adjacent normal colon tissue. The pathological characteristics of colon adenocarcinoma (a) and adjacent normal colon tissue (c) in the tumor specimen are showed in hematoxylin and eosin staining slide. The density of ASS protein expression in colon adenocarcinoma (b) and adjacent normal colon tissue (d) is showed in the image of immunohistochemistry with ASS antibody.
(TIF)

Figure S2 **Strong expression of ASL in colon carcinoma tissue as determined by immunohistochemical staining.** The data shown are from matched tissue specimens: (a and b) adjacent normal colon tissue, (c and d) cancer tissue. Same sample showed in 10 times amplification (a,c) and 20 times amplification (b,d). The density of ASL protein expression in colon adenocarcinoma (c,d) and adjacent normal colon tissue (a,b) is showed in the image of immunohistochemistry with ASL antibody.
(TIF)

Table S1 **Primer sequences used for quantitative PCR analysis of CATs.**
(DOC)
