# Supplementary figures and images for: Overexpression of Arginine Transporter CAT-1 Is Associated with Accumulation of L-Arginine and Cell Growth in Human Colorectal Cancer Tissue
Source: PLoS One. 2013 Sep 6;8(9):e73866. doi: 10.1371/journal.pone.0073866 (PMC3765253; doi:10.1371/journal.pone.0073866)

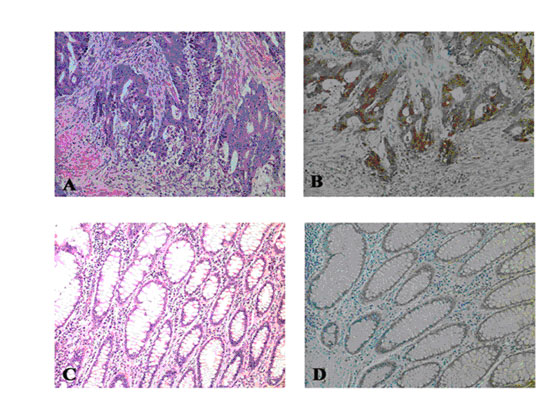

Supplement: Figure S1 — Strong expression of ASS in colon carcinoma tissue as determined by immunohistochemical staining. The samples are from matched tissue specimens: (a and b) cancer tissue, (c and d) adjacent normal colon tissue. The pathological characteristics of colon adenocarcinoma (a) and adjacent normal colon tissue (c) in the tumor specimen are showed in hematoxylin and eosin staining slide. The density of ASS protein expression in colon adenocarcinoma (b) and adjacent normal colon tissue (d) is showed in the image of immunohistochemistry with ASS antibody. (TIF) [file pone.0073866.s001.tif]

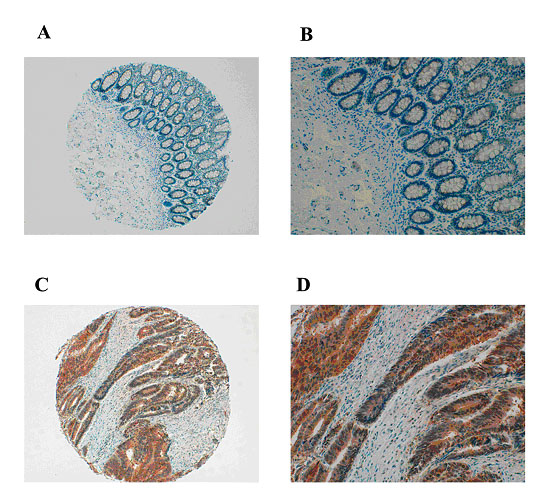

Supplement: Figure S2 — Strong expression of ASL in colon carcinoma tissue as determined by immunohistochemical staining. The data shown are from matched tissue specimens: (a and b) adjacent normal colon tissue, (c and d) cancer tissue. Same sample showed in 10 times amplification (a,c) and 20 times amplification (b,d). The density of ASL protein expression in colon adenocarcinoma (c,d) and adjacent normal colon tissue (a,b) is showed in the image of immunohistochemistry with ASL antibody. (TIF) [file pone.0073866.s002.tif]
