# Supplementary material for: Overexpression of Arginine Transporter CAT-1 Is Associated with Accumulation of L-Arginine and Cell Growth in Human Colorectal Cancer Tissue
Source: PLoS One. 2013 Sep 6;8(9):e73866. doi: 10.1371/journal.pone.0073866 (PMC3765253; doi:10.1371/journal.pone.0073866)
Supplement: Table S1 — Primer sequences used for quantitative PCR analysis of CATs. (DOC) [file pone.0073866.s003.doc]

Table S1. Primer sequences used for quantitative PCR analysis of CATs.

| ID | **Primer name** | **Sequence (5'-3')** | **No. of NTs** |
| --- | --- | --- | --- |
| 1 | Homo-GAPDH-primer-upper | GAGTCAACGGATTTGGTCGTATTG | 24 |
| 2 | Homo-GAPDH-primer-lower | CTCCTGGAAGATGGTGATGGGAT | 23 |
| 3 | SLC7A1(hCAT1)primer-upper | ATGGGTGGAAACGCTGATGATAC | 24 |
| 4 | SLC7A1(hCAT1)primer-lower | ACCTTGCCTGTTAAGTCTGGGTG | 23 |
| 5 | SLC7A2(hCAT-2A)primer-upper | TTTAACACTTATGATGCCGTACTACCT | 27 |
| 6 | SLC7A2(hCAT-2A)primer-lower | GCAACTGGTGACTGCCTCTTACT | 23 |
| 7 | SLC7A2(hCAT-2B)primer-upper | ATGCCTCGTGTAATCTATGCTATG | 24 |
| 8 | SLC7A2(hCAT-2B)primer-lower | ACTGCACCCGATGATAAAGTAGC | 23 |
| 9 | SLC7A4-primer-upper | GCTGCGTGCTTGTCTTTGGGAACT | 24 |
| 10 | SLC7A4-primer-lower | TTCAGCATGAGGCAGATGTTGAGG | 24 |
| 11 | SLC3A2-primer-upper | GTTGCGCAGACTGGTCTCGAACT | 23 |
| 12 | SLC3A2-primer-lower | TGGCTCATGGTGCCTGTAACCTC | 23 |
| 13 | SLC7A6(Y+LAT2)-primer-upper | TTCTTGACAGGCAGTGGCGTGAT | 23 |
| 14 | SLC7A6(Y+LAT2)-primer-lower | CTGGGACTGGCTGGTGTTAGGGA | 23 |
| 15 | SLC7A7(y+LAT1)-primer-upper | CATCATCAGAGTGCCAGAACATAA | 24 |
| 16 | SLC7A7(y+LAT1)-primer-lower | TAGCTCTAGCCAGTAGACCAGAAAC | 25 |
